# Benefits of Regular Exercise on Inflammatory and Cardiovascular Risk Markers in Normal Weight, Overweight and Obese Adults

**DOI:** 10.1371/journal.pone.0140596

**Published:** 2015-10-16

**Authors:** Olivia Santos Gondim, Vinicius Tadeu Nunes de Camargo, Fernanda Almeida Gutierrez, Patricia Fátima de Oliveira Martins, Maria Elizabeth Pereira Passos, Cesar Miguel Momesso, Vinicius Coneglian Santos, Renata Gorjão, Tania Cristina Pithon-Curi, Maria Fernanda Cury-Boaventura

**Affiliations:** 1 Institute of Physical Activity and Sports Sciences, Post-Graduate Program in Human Movement Science, Cruzeiro do Sul University, São Paulo, SP, Brazil; 2 Department of Physiology and Biophysics, Institute of Biomedical Sciences, University of São Paulo, SP, Brazil; Dasman Diabetes Institute, KUWAIT

## Abstract

Obesity is a worldwide epidemic that increases the risk of several well-known co-morbidities. There is a complicated relationship between adipokines and low-grade inflammation in obesity and cardiovascular disease (CVD). Physical activity practices have beneficial health effects on obesity and related disorders such as hypertension and dyslipidemia. We investigated the effects of 6 and 12 months of moderate physical training on the levels of adipokines and CVD markers in normal weight, overweight and obese volunteers. The 143 participants were followed up at baseline and after six and twelfth months of moderate regular exercise, 2 times a week, for 12 months. The volunteers were distributed into 3 groups: Normal Weight Group (NWG,), Overweight Group (OVG) and Obese Group (OBG). We evaluated blood pressure, resting heart rate, anthropometric parameters, body composition, fitness capacity (VO_2max_ and isometric back strength), cardiovascular markers (CRP, total cholesterol, LDL-c, HDL-c, homocysteine) and adipokine levels (leptin, adiponectin, resistin, IL-6 and TNF-alpha). There were no significant changes in anthropometric parameters and body composition in any of the groups following 6 and 12 months of exercise training. Leptin, IL-6 levels and systolic blood pressure were significantly elevated in OBG before the training. Regular exercise decreased HDL-c, leptin, adiponectin and resistin levels and diastolic blood pressure in OVG. In OBG, exercise diminished HDL-c, homocysteine, leptin, resistin, IL-6, adiponectin. Moderate exercise had no effect on the body composition; however, exercise did promote beneficial effects on the low-grade inflammatory state and CVD clinical markers in overweight and obese individuals.

## Introduction

Obesity is a public health problem and can be classified as a world epidemic that leads to an elevation in medical costs. Obesity and overweight have been shown to increase the risk of several well-known co-morbidities such as cardiovascular disease. Obesity is characterized by excess energy intake resulting in an expansion of adipose tissue depots, visceral adiposity, hypertrophy, hyperplasia, and adipocyte dysfunction [1]. In obese individuals there are marked increases in the secretion of pro-inflammatory adipokines including leptin, resistin, IL-6 and TNF-alpha, and decreased production of anti-inflammatory adipokines such as adiponectin. This change in adipokine balance is a key component of pathogenic metabolic and immune responses and has impacts on angiogenesis, blood pressure and lipid metabolism, all of which are linked with cardiovascular disease [2, 3]

Adipokines exert their effects in a paracrine or endocrine manner, inducing hypertension and dyslipidemia through an elevation in mineralocorticoid and catecholamine activity and unbalanced lipid metabolism. Leptin plays important roles in the regulation of food intake, energy expenditure, metabolism, the neuroendocrine axis, and immune function [1, 4]. There is strong evidence that leptin levels are positively correlated with BMI and adiposity and that obese individuals are leptin resistant [1, 3]. Elevation of serum resistin levels in obesity also has been reported by several groups and is associated with metabolic syndrome, dyslipidemia and inflammatory markers [5–7]. IL-6 is a novel adipokine expressed by both adipocytes and the stromovascular matrix of visceral white adipose tissue implicated in the genesis of obesity. Plasma IL-6 concentration increases with weight gain and decreases upon weight loss [1, 3]. IL-6, which affects appetite and energy intake through actions in the hypothalamus, is also a major determinant of the hepatic production of CRP and is capable of suppressing lipoprotein lipase activity [1, 8, 9]. In contrast, adiponectin has a cardiovascular protective role and has been proposed to serve as a link between obesity and cardiovascular disease. Adiponectin predominantly affects the liver, skeletal muscle, vascular wall, and endothelial cells and has anti-inflammatory and anti-atherogenic effects [10].

There is a complicated relationship between these adipokines and low-grade inflammation and/or sympathetic regulation during obesity and cardiovascular disease [3, 9, 10]. Alternative approaches that affect the early developmental phases of cardiovascular disease might show a greater impact [11–13]. Physical activity has been postulated to have beneficial health effects, and thus may potentially be used therapeutically for obesity and related disorders such as hypertension and dyslipidemia. Exercise could act by improving adipokine profiles and cardio metabolic and clinical markers [11–16]; however, the role of exercise on lipid metabolism and inflammatory markers remains inconclusive due the several drawbacks around the intensity and frequency of training, target group and BMI levels in the studies.

We hypothesized that moderate physical exercise could improve clinical markers of cardiovascular disease, mainly in overweight and obese patients, and that this effect may be mediated by a reduction of adipokines and inflammation. Therefore, we investigated the effects of 6 and 12 months of moderate physical training on adipokines levels, cardiometabolic and clinical markers in normal weight, overweight and obese volunteers.

## Materials and Methods

### Participants

Six hundred and fifty-seven participants were recruited at the beginning of the intervention from the Institute of Physical Activity and Sport Sciences of the Cruzeiro do Sul University. One hundred and forty-three volunteers completed the 12-month exercise intervention, including 29 males and 114 females, with a mean age of 56 ± 10 years old. The participants were excluded from the study if they did not obtain at least 80% attendance in the activities; did not participate in the 3 steps proposed by our study (at baseline and after six and twelve months of regular exercise); and were not fasted for 8 hours prior to blood collection. Screening and recruitment occurred between March 2012 and March 2014. All of the practitioners of regular exercise were participants in a structured physical activity program at community-based health and fitness center at the Institute of Physical Activity and Sport Sciences of the Cruzeiro do Sul University. Written informed consent was signed by all of the participants before being enrolled into the study. The study was approved by the Human Ethics Committee from Cruzeiro do Sul University (number CS/UCS-027/2012), Sao Paulo, Brazil.

The participants were evaluated at baseline and after 6 and 12 months of regular exercise. We compared data from the same individual in different time points to determine training effect and minimize group heterogeneity. The volunteers were distributed into the following 3 groups according to body mass index (BMI): Normal Weight Group (NWG, BMI < 25 kg/m^2^), Overweight Group (OVG, BMI ≥ 25 kg/m^2^ and <30 kg/m^2^), and Obese Group (OBG, BMI ≥ 35 kg/m^2^). The gender distribution was similar among the NWG (87% female; 13% male), OVG (80% female; 20% male) and OBG (79% female; 21% male). On the experimental day, the subjects reported to the Sports Center of the Institute of Physical Activity and Sports Sciences at 08.00 h after an overnight fast and 24 hours of rest. The participants filled out a questionnaire that included questions regarding personal characteristics, several chronic diseases, medications, health, smoking and physical training history. Heart rate (HR, Polar FT7, Kempele, Finland) and manual blood pressure were obtained using an aneroid sphygmomanometer and cuff (Premium, Sao Paulo, Brazil) after the participant had been seated for at least 5 minutes. Then, the anthropometric parameters, body composition, blood collection and fitness capacity were measured.

Of the 143 participants, 46% reported dyslipidemia, 46% reported hypertension and 19% reported Diabetes Mellitus (DM). The prevalence of dyslipidemia, hypertension and DM were greater in the OBG (54%, 53% and 26%, respectively) compared to OVG (39%, 45% and 17%, respectively) and NWG (34%, 19% and 9%, respectively) (p<0.05), as expected. With respect to medication, 16% have previously received a prescription for dyslipidemia, 37% for antihypertension and 11% for DM. The diet of the volunteers was not controlled in this study. Brazilian population food intake is based on approximately 69.5% daily energy consumption being provided by natural or minimally processed foods, 21.5% by ultra-processed foods and 9.0% by processed foods. This ultra-processed food consumption contributes to the Brazilian food intake characteristic described as a diet with higher levels of energy density, total fat, saturated fat and trans fat, sucrose and sodium and insufficient fiber content (Louzana et al., 2015; Sarno et al., 2013).

### Anthropometry and body composition

Anthropometric measures including height, body mass, abdomen circumference, waist circumference (WC) and hip circumference (HC) were evaluated using standard techniques [17]. BMI, waist/hip ratio (WC/HC) and waist/height ratio (WC/height) were calculated. Then, body composition (percentage of total body fat, lean mass and fat mass) was assessed by bioimpedance analysis (*Biodynamics Corporation*, USA; 310e).

### The training program

The volunteers underwent a supervised twelve-month exercise program consisting of 60 minutes of exercise 2 times a week. The training sessions were supervised by qualified trainers in the modalities swimming (41 volunteers) and/or water aerobics (106 volunteers), all performed in the Sports Center of the Institute of Physical Activity and Sports Sciences. During the training sessions, the first 5 minutes were used for a warm-up, the next 40 minutes included aerobic exercises interspersed with resistance exercises and the final 5 minutes were reserved for stretching. HR (Polar FT7, Kempele, Finland) was recorded during sessions for the calculation of the mean HR, and the HRmax (220 –age) was obtained to estimate training load. The mean training intensity was 66 ± 10% of age predicted by the HRmax.

### Blood sampling

Blood samples for biochemical and hormonal measurements were withdrawn after 8 h of fasting and 24 h after the last exercise session. Blood samples (20 ml, 10 ml EDTA tube and 10 ml dry tube) were taken from the antecubital vein before the training period, and six months and 12 months after the training began. Serum and plasma were separated by centrifugation for 10 min at 1000 × g and were subsequently stored in aliquots at −80°C until further analysis.

### Biochemical measurements

Adipokines and homocysteine were measured using an enzyme-linked immunosorbent assay (Quantikine ELISA) method (R & D System, Minneapolis, USA). A monoclonal antibody specific for each molecule has been pre-coated onto a microplate. After incubation with standards and samples and washing away any unbound substances, an enzyme-linked monoclonal antibody specific for each adipokine and homocysteine was added. Following another wash, a substrate solution was added to the wells and color developed in proportion to the adipokines and homocysteine levels. The color development was stopped and the intensity of color was measured at 450 nm using a spectrophotometer (SpectraMax Plus, Molecular Devices, California, USA) The sensitivity was 9.4 pg/mL, 15.6 pg/mL, 0.026 ng/mL, 3.9 ng/mL and 0.195 nmol/mL for IL-6, leptin, resistin, adiponectin and homocysteine, respectively. CRP measurement was performed using a latex-enhanced immunoturbidimetric assay capable of measuring protein levels both within and outside the normal range (Bioclin Diagnostic, *Minas Gerais*, *Brazil)*. The CRP concentration was determined in duplicate by measuring the optical density at 505 nm. The intra-assay CV was 1.6%-4%, the inter-assay CV was 0.6%-1.5%, and the sensitivity for CRP was 0.2962 mg/L. The lipid profile (total cholesterol, LDL-c and HDL-c) was determined using a colorimetric enzymatic method (Bioclin Diagnostic*)*. All of the above assays were carried out according to the manufacturer’s instructions. The total cholesterol, LDL-c and HDL-c were consumed by cholesterol esterase and cholesterol oxidase in the reaction and in the presence of enzyme and a chromogenic reagent, a color was formed in proportion to the amount of total cholesterol, LDL-c and HDL-c in the sample. The reading was performed at 500 nm using a spectrophotometer (SpectraMax Plus, Molecular Devices), and the results are expressed in mg/dL. The concentration of total cholesterol, HDL-c and LDL-c was calculated from the ratio between the absorbance of the sample and the standard absorbance in a spectrophotometer multiplied by standard concentration.

### Fitness Capacity

Fitness capacity tests were performed at baseline, after 6 months and after 12 months of training to detect physiological adaptation that might provide objective evidence of adherence to the exercise prescription. The isometric back strength was evaluated using the protocol of Johnson and Nelson (1979) [18]. A measurement of aerobic capacity consisted of a 12-minute run-walk test [19]. The volunteers were instructed to run or walk the maximum tolerated distance in 12 minutes. The subjects ran in a group to provide a source of motivation and were vigorously encouraged during the test. Based on the measured distance, an estimate of VO_2max_ in mL/(kg·min) was calculated: VO_2max_ = [(distance – 504.9)/44.73].

### Statistical method

Sample Size was calculated based on confidence interval of 95% and confidence level of 10%. Statistical analysis was performed using GraphPad Prism 5 *(Graph Pad Software*, *USA)* and the values are presented as the mean and standard error of the mean. The comparison of the values of biochemical data, anthropometric data and body composition were calculated using a Two-way ANOVA method with repeated measures (different time points: 0, 6 and 12 months) followed by Bonferroni test. Statistical significance was considered for *P* values less than 0.05.

## Results

The participant characteristics are presented in **Table 1**. As expected, the total body mass, fat and lean mass were higher in OBG and OVG than NWG. OBG and OVG were older than NWG. In this study, the age was positively correlated with the % of fat mass (r = 0.19, p = 0.021); waist (r = 0.19, p = 0.021); waist hip rate (r = 0.24, p = 0.003) and leptin levels (r = 0.19, p = 0.034). There were no significant changes in the anthropometric parameters (BMI, waist, waist/hip ratio, waist/height ration) and body composition (total body mass, fat mass, lean mass) in any of the groups following 6 and 12 months of exercise training (**Tables 2 and 3**). After 6 months of exercise we observed a reduction in the percentage of body fat mass by 5.07% in the NWG only (**Tables 2 and 3**).

**Table 1:** General characteristic of the participants prior to training.

	NWG	OVG	OBG	p
**Age (years)**	47 ±18	58 ±13 ^a^	58 ±12 ^a^	<0.01
**Height (cm)**	1.60 ± 0.02	1.59 ± 0.05	1.59 ± 0.01	ns
**Weight (kg)**	58 ± 3	70 ± 5 ^a^	86 ± 5 ^a^ ^b^	<0.001
**BMI (kg/m2)**	22 ±0.4	27 ± 0.1 ^a^	34 ± 3 ^a^ ^b^	<0.001
**Lean mass (kg)**	42 ± 1	47 ± 3 ^a^	53 ± 1 ^a^ ^b^	<0.001
**Fat Mass (kg)**	16 ± 2	24 ± 3 ^a^	33 ± 4 ^a^ ^b^	<0.001
**% of fat mass**	28 ± 2	34 ± 1	38 ± 1	ns
**VO**_**2max**_ **(ml/kg/min)**	19.15 ± 1.52	15.63 ± 0.71	13.04 ± 0.78 ^a^	<0.001

The values presented are the mean ± standard error of mean for 32 normal weight (NWG), 59 overweight (OVG) and 52 obese (OBG) individuals.

^a^ comparison to NWG

^b^ comparison to OVG.

**Table 2:** Effect of exercise on anthropometric parameters of normal weight (NWG), overweight (OVG) and obese (OBG) groups.

	NWG	OVG	OBG	p
**Weight (kg)**				
**0**	58 ± 3	70 ± 5 ^a^	86 ± 5 ^a^ ^b^	<0.001
**6**	58 ± 4	70 ± 6 ^a^	86 ± 8 ^a^ ^b^	<0.001
**12**	59 ± 6	70 ± 6 ^a^	85 ± 12 ^a^ ^b^	<0.001
**p**	ns	ns	ns	
**BMI (kg/m** ^**2**^ **)**				
**0**	22 ± 0	27 ± 0 ^a^	34 ± 3 ^a^ ^b^	<0.001
**6**	22 ± 1	27 ± 0 ^a^	34 ± 4 ^a^ ^b^	<0.001
**12**	23 ± 2	27 ± 0 ^a^	34 ± 5 ^a^ ^b^	<0.001
**p**	ns	ns	ns	
**Lean mass (kg)**				
**0**	42 ± 1	47 ± 3 ^a^	53 ± 1 ^a^ ^b^	<0.05
**6**	43 ± 3	46 ± 3	52 ± 4 ^a^ ^b^	<0.01
**12**	42 ± 2	46 ± 4	53 ± 5 ^a^ ^b^	<0.01
**p**	ns	ns	ns	
**Fat Mass (kg)**				
**0**	16 ± 2	24 ± 3 ^a^	33 ± 4 ^a^ ^b^	<0.001
**6**	15 ± 1	24 ± 3 ^a^	33 ± 3 ^a^ ^b^	<0.001
**12**	16 ± 3	24 ± 2 ^a^	33 ± 7 ^a^ ^b^	<0.001
**p**	ns	ns	ns	
**% of fat mass**				
**0**	28 ± 2	34 ± 1 ^a^	38 ± 1 ^a^ ^b^	<0.001
**6**	27 ± 2	34 ± 1 ^a^	38 ± 1 ^a^ ^b^	<0.001
**12**	28 ± 2	34 ± 1 ^a^	38 ± 1 ^a^ ^b^	<0.001
**p**	ns	ns	ns	

The values presented are the mean ± standard error of mean for 32 normal weight, 59 overweight and 52 obese.

^a^ comparison to NWG

^b^ comparison to OVG.

**Table 3:** Effect of exercise on anthropometric parameters of normal weight (NWG), overweight (OVG) and obese (OBG) groups.

	NWG	OVG	OBG	p
**Waist (cm)**				
**0**	78 ± 1	88 ± 1 ^a^	100 ± 2 ^a^ ^b^	<0.001
**6**	76 ± 1	87 ± 1 ^a^	99 ± 1 ^a^ ^b^	<0.001
**12**	78 ± 1	88 ± 1 ^a^	101 ± 2 ^a^ ^b^	<0.001
**p**	ns	ns	ns	
**Waist/Hip rate**				
**0**	0.83 ±0.01	0.86 ±0.009	0.89 ± 0.01 ^a^	<0.001
**6**	0.82 ±0.01	0.86 ±0.009	0.90 ± 0.01 ^a^ ^b^	<0.01
**12**	0.83 ±0.01	0.86 ±0.008	0.91 ± 0.01 ^a^ ^b^	<0.01
**p**	ns	ns	ns	
**Waist/Height rate**				
**0**	0.48 ± 0.01	0.55 ± 0.01 ^a^	0.63 ± 0.01 ^a^ ^b^	<0.001
**6**	0.48 ± 0.01	0.55 ± 0.01 ^a^	0.63 ± 0.01 ^a^ ^b^	<0.001
**12**	0.48 ± 0.01	0.54 ± 0.01 ^a^	0.63 ± 0.01 ^a^ ^b^	<0.001
**p**	ns	ns	ns	

The values presented are the mean ± standard error of mean for 32 normal weight, 59 overweight and 52 obese.

^a^ comparison to NWG

^b^ comparison to OVG.

**Fig 1:**
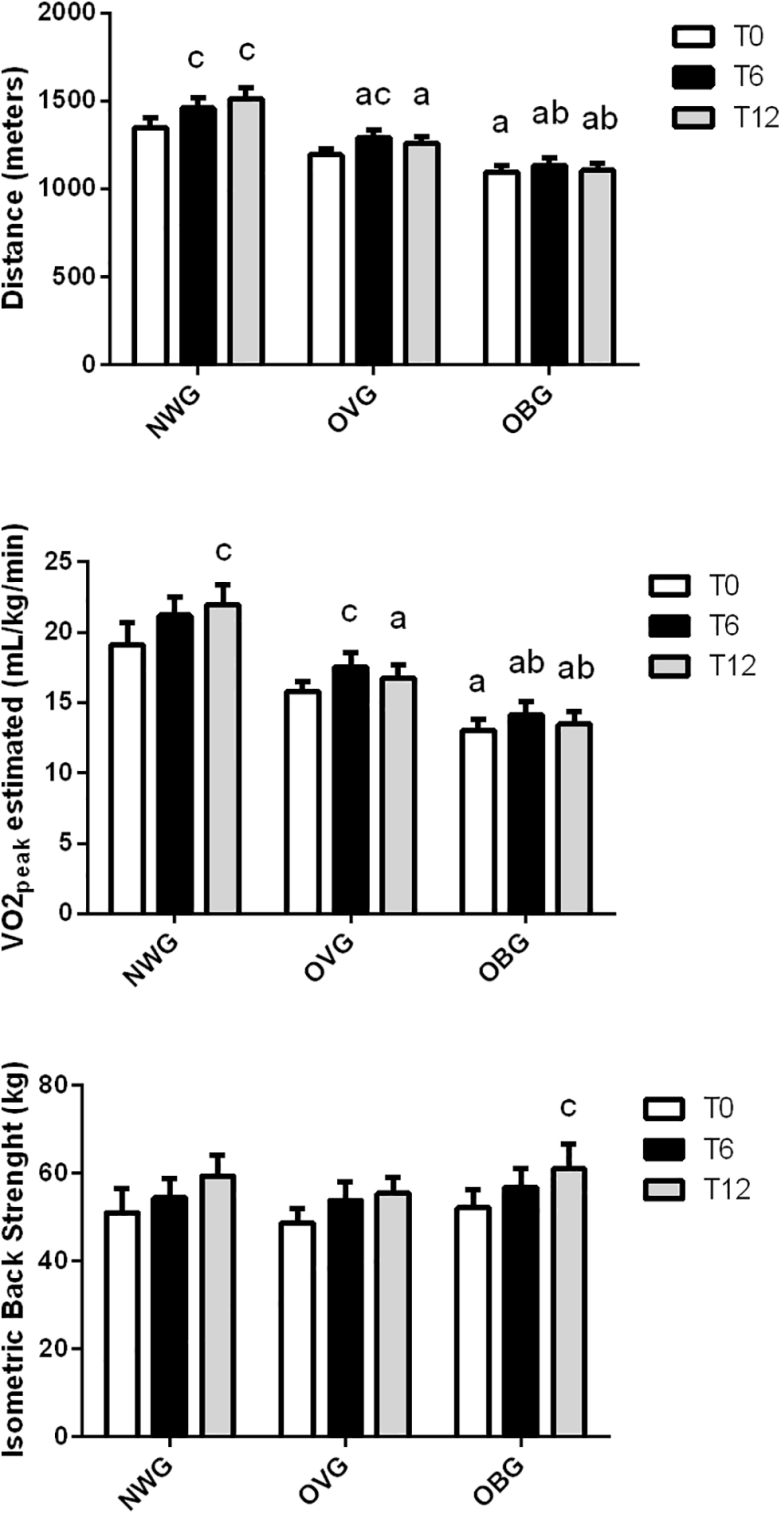
Effect of exercise on fitness capacity in normal weight (NWG), overweight (OVG) and obese (OBG) groups. Aerobic capacity and isometric back strength were evaluated before (T0), after 6 months (T6) and 12 months (T12) of the exercise training program. The values presented are the mean ± standard error of mean. ^c^ comparison to before training.

However, moderate exercise improved the distance of the aerobic capacity test in OBG after 6 and 12 months and in OVG after 6 months; and VO_2peak_ estimated in OBG after 12 months and in OVG after 6 months (**Fig 1**). The aerobic capacity was lower in OBG compared to NWG and OVG after 6 and 12 months (**Fig 1**). In OBG, lower limb strength improved (from 49 ± 2 to 58 ± 3 kg, p < 0.001) after 12 months of exercise (**Fig 1**).

Considering all of the participants, the leptin levels were positively correlated with age (r = 0.19, p<0.05), weight (r = 0.32, p<0.0001), waist (r = 0.44, p<0.0001), hip (r = 0.61, p<0.0001) and abdomen circumferences (r = 0.51, p<0.0001), adiponectin (r = 0.3, p<0.001) and IL-6 (r = 0.25, p<0.05), and negatively correlated with VO_2peak_ estimated (r = -0.32, p<0.0001), and isometric back strength (r = -0.18, p<0.05). Adiponectin had a positive correlation with total cholesterol (r = 0.2, p<0.05), LDL-c (r = 0.19, p<0.05), CRP (r = 0.23, p<0.05) and a negative correlation with HDL-c (r = -0.32, p<0.001).

**Fig 2:**
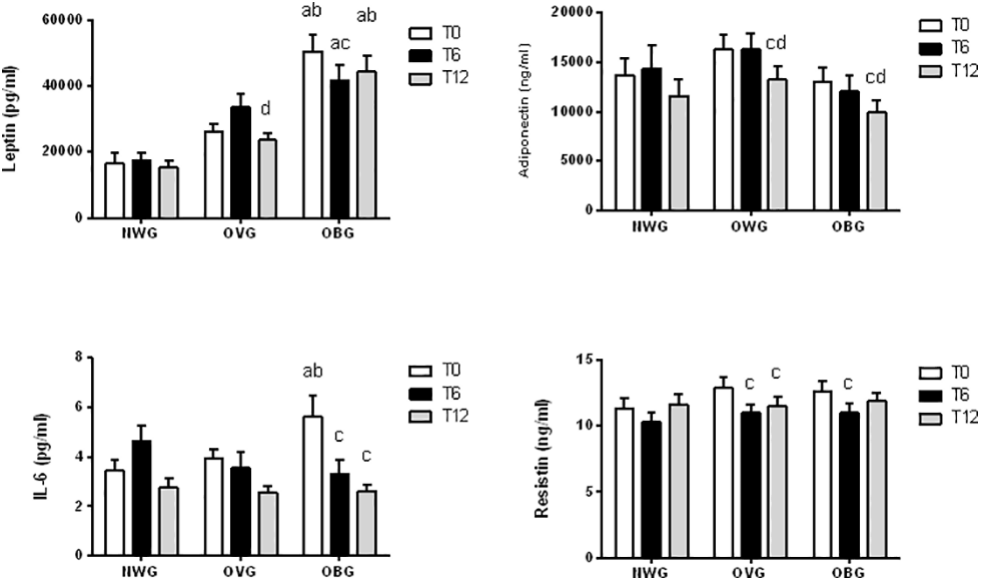
Effect of exercise on adipokines in normal weight (NWG), overweight (OVG) and obese (OBG) groups. Plasma and serum was separated immediately after blood collection before (T0), after 6 months (T6) and 12 months (T12) of the exercise training program. The plasma or serum concentrations of leptin, adiponectin, IL-6 and resistin were determined using an Enzyme-linked immunosorbent assay (ELISA) method. The values presented are the mean ± standard error of mean. ^a^ comparison to normal weight group; ^b^ comparison to overweight group; ^c^ comparison to before program training (T0); ^d^ comparison to after 6 months of program training.

Leptin and IL-6 levels were significantly elevated in OBG before the training (**Fig 2**). The chronic exercise had no effect on adipokine levels in NWG; however, training decreased leptin and adiponectin levels (by approximately 34% and 15%, respectively) after 12 months and resistin by 15% after 6 and 12 months in OVG. In OBG, the exercise diminished the leptin and resistin concentration by 12–18% after 6 months, IL-6 after 6 and 12 months (by 41–53%) and adiponectin by 18% after 12 months (**Fig 2**).

In comparison to baseline, the cardiometabolic markers including total cholesterol, LDL-c and CRP levels remained unchanged following chronic exercise in the groups. After 12 months of training we observed an approximately 10% decrease in the HDL-c levels in OVG and OBG, and a 27% decrease in homocysteine in OBG (**Table 4**).

**Table 4:** Effect of exercise on cardiometabolic parameters in normal weight (NWG), overweight (OVG) and obese (OBG) groups.

	NWG	OVG	OBG	p
**Total Cholesterol (mg/dL)**				
**0**	177 ± 7	178 ± 6	186 ± 7	ns
**6**	185 ± 10	184 ± 7	186 ± 6	ns
**12**	178 ± 7	180 ± 6	186 ± 5	ns
**p**	ns	ns	ns	
**LDL-c (mg/dL)**				
**0**	83 ± 5	79 ± 3	82 ± 3	ns
**6**	86 ± 5	82 ± 4	85 ± 4	ns
**12**	83 ± 5	79 ± 4	83 ± 4	ns
**p**	ns	ns	ns	
**HDL-c (mg/dL)**				
**0**	55 ± 4	61 ± 4	58 ± 3	ns
**6**	58 ± 5	58 ± 4	60 ± 3	ns
**12**	55 ± 4	55 ± 4 ^c^	55 ± 4 ^d^	ns
**p**	ns	<0.01	<0.05	
**CRP (mg/L)**				
**0**	0.7 ± 0.09	0.8 ± 0.09	1.0 ± 0.10	ns
**6**	0.7 ± 0.11	0.9 ± 0.11	1.3 ± 0.23 ^a^	<0.05
**12**	0.5 ± 0.07	0.8 ± 0.13	1.1 ± 0.16 ^a^ ^b^	<0.05
**Homocysteine (nmol/mL)**				
**p**	ns	ns	ns	
**0**	2.4 ± 0.6	2.7 ± 0.3	3.2 ± 0.5	ns
**6**	1.4 ± 0.04	2.1 ± 0.3	2.8 ± 0.5	ns
**12**	1.6 ± 0.4	2.6 ± 0.4	2.3 ± 0.3 ^c^	ns
**p**	ns	ns	<0.05	

The values presented are the mean ± standard error of mean for 27 normal weight, 57 overweight and 52 obese individuals.

^a^ comparison to NWG

^b^ comparison to OVG

^c^ comparison to before program training (T0)

^d^ comparison to after 6 months program training (T6).

**Fig 3:**
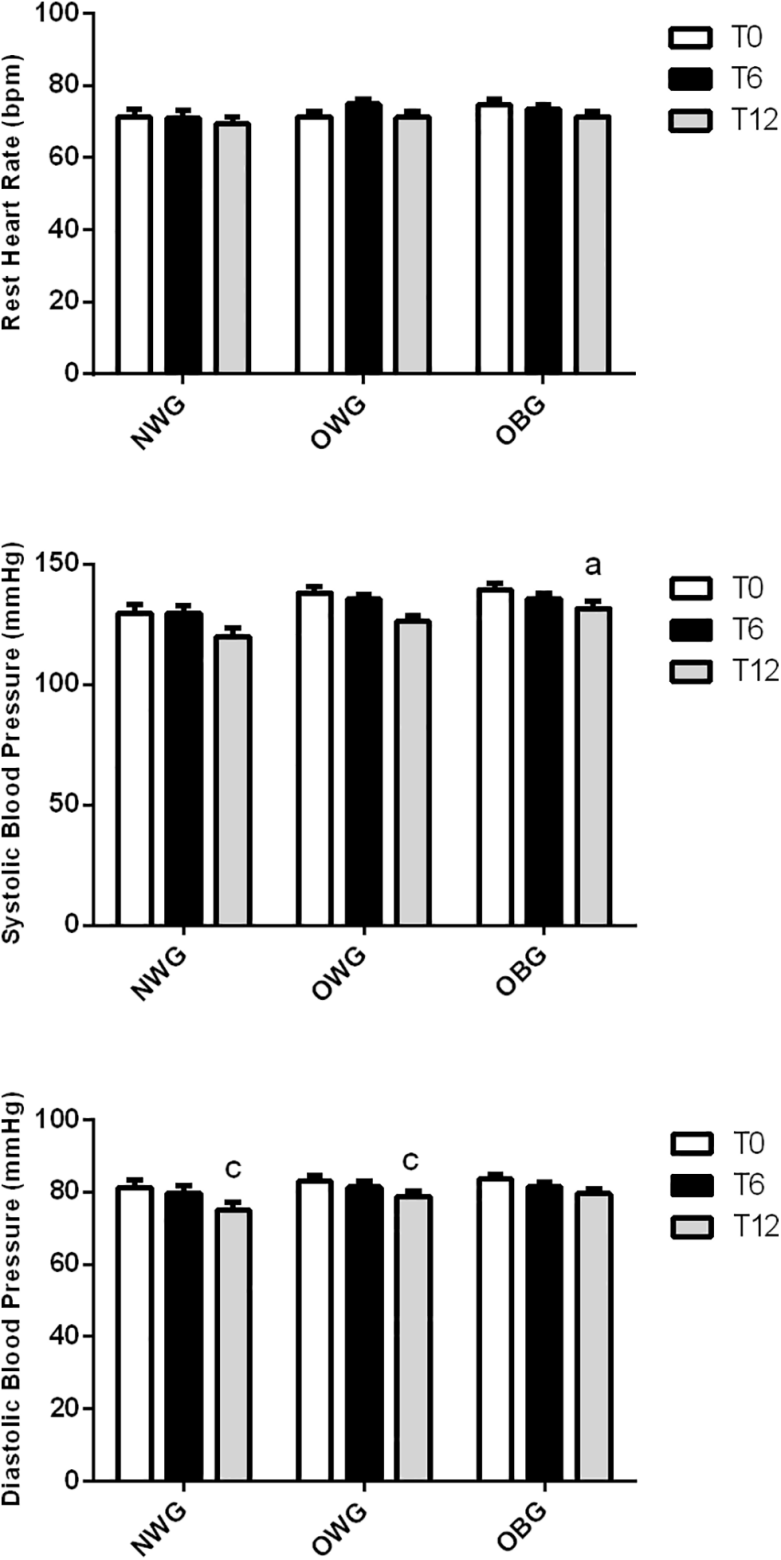
Effect of exercise on blood pressure and resting heart rate in normal weight (NWG), overweight (OVG) and obese (OBG) groups. The systolic and diastolic blood pressure was evaluated before (T0), after 6 months (T6) and 12 months (T12) of the exercise training program. The values presented are the mean ± standard error of mean. ^a^ comparison to normal weight group; ^c^ comparison to before training.

The resting heart rate and systolic blood pressure were not significantly different from those observed following 6 and 12 months of training. Systolic blood pressure was 10% higher in OBG than in NWG. The physical activity reduced the diastolic blood pressure by 5–10% 12 months of training in NWG and OVG (**Fig 3**).

## Discussion

In the present study, the moderate exercise program improved the low-grade inflammatory state and cardiovascular system, reducing leptin and resistin levels and diastolic blood pressure in OVG, and leptin, resistin, IL-6 and homocysteine levels in OBG; however, body composition and some cardiometabolic markers that are more related to diet habits remained unchanged or worsened (HDL-c and adiponectin). We suggest that the control of energy balance through diet and physical activity are crucial to improve these markers and decrease cardiovascular risk.

The lack of diet control was a limitation in our study. We estimated energy expenditure to be around 200 to 400 kcal per activity, i.e. 400 to 800 kcal per week, which should contribute to loss of 1 kg in approximately 11–12 weeks. However, the lack of effect on body composition could be explained by: the easy replacement of food intake; and/or, in a long run, the adaptations to exercise decreased the energy expenditure during the exercise; and/or the low frequency of exercise.

In the lipostatic theory of body-weight set point, the release of leptin from adipocytes is a negative feedback loop to suppress appetite and prevent weight gain. Leptin acts in the hypothalamic arcuate nucleus and tractus solitarii and is responsible for controlling glucose homeostasis, energy balance and SNS activation [3]. The peripheral function of leptin involves angiogenesis, hematopoiesis, bone density, wound healing, the immune system, energy metabolism regulation, and nutrient intestinal absorption [20].

Although the circulating leptin levels are very high in obese subjects, leptin is unable to effectively prevent weight gain, defined as leptin resistance, ant this was observed in our study [2].

Thereby, these studies support that several mediators of inflammation are related with hyperleptinemia and leptin resistance development in states of obesity induced by diet. The activation of inflammatory signaling initiates the development of leptin resistance and up regulates pro inflammatory cytokines such as IL-6 [20]. We observed that leptin had a positive correlation with IL-6. Huang et al. (2014) recently demonstrated that mononuclear cells (PBMCs) are more stimulated by LPS, resulting in elevated IL-6 production in obese individuals [21]. We also observed that leptin had a positive correlation with IL-6 and elevated levels of these adipokines in the obese group before training. Higher levels of resistin are also reported in obese patients who have elevated levels of inflammatory markers [2]; however, we did not observe a higher resistin concentration in OBG in our study.

Evidence suggests that the protective effect of exercise may, to some extent, be ascribed to the anti-inflammatory effect of regular exercise mediated via a reduction in visceral fat mass and/or by induction of an anti-inflammatory environment, increasing IL-10 and IL1-ra, with each bout of exercise [22]. We suggested that these anti-inflammatory properties may improve adipocytokine levels. Khadir et al. (2014) reported that physical exercise may attenuate the expression of Dual specificity protein phosphatase 1 (DUSP1), involved in energy expenditure, with an increase in the expression of PGC-1α and a reduction in JNK and ERK activities along with an attenuated inflammatory response [14]. The anti-inflammatory exercise effects may be associated with the down-regulation of NADPH oxidase, ERK1/2 and SAPK/JNK activities, and increased SOD-1 expression [15]. In our study we observed a decrease on leptin, IL-6 and resistin after regular exercise in the overweight and obese groups, independent of weight loss. The same effect on IL-6 levels was observed by other researchers [12, 16] in overweight and obese indigenous Australian men; on leptin and IL-6 levels in obese adults [23]; and on leptin, IL-6 and resistin concentrations in overweight and obese children [24].

Leptin levels after short-term training appear to be well correlated with the amount of work performed and dissociated from a concomitant decrease of fat mass [11]. Metabolic adaptation was also correlated with the decrease in circulating leptin following calorie restriction plus vigorous exercise [25]. The individuals with higher aerobic capacity lost more weight and have lower changes in leptin and insulin concentrations, suggesting better metabolic flexibility [26]. In obese patients, IL-6 acts predominantly as a pro-inflammatory cytokine; however, the identification of IL-6 as a myokine provides a contrasting and hence paradoxical identity as an anti-inflammatory cytokine. Exercise enhances muscle-derived IL-6, which is in correlation with muscle mass and exercise intensity and stimulates the release of other anti-inflammatory cytokines including IL-10 and IL-1ra [27]. A previous study suggested that physical exercise reduced the expression of various HSPs with concomitant attenuation in the endogenous levels of IL-6 [28].

Plasma resistin was correlated to cardiovascular and pro-inflammatory markers and several components of the metabolic syndrome in obese adolescents [29]. In our study, the plasma resistin concentration diminished after the training program, similar to a previous study conducted during 8 weeks of training in obese students [30] and during 7 months of intense exercise associated with caloric restriction in morbidly obese individuals [31].

Adiponectin inhibits interleukin-6 (IL-6) production accompanied by the induction of the anti-inflammatory cytokines IL-10 and IL-1 receptor antagonist in human macrophages, and has paracrine effects on endothelial progenitor cells (EPCs), endothelial cells (ECs), vascular smooth muscle cells (VSMCs), cardiomyocytes (CMs), and cardiac fibroblasts (CFs) [2]; however, the role of adiponectin in cardiovascular risk remains inconclusive due to differences among reports including variations in populations, confounding factors and different isoforms of adiponectin [32]. Several studies have demonstrated that adiponectin levels increase after a training program in healthy and obese women [13, 33]. Zhang et al., 2014 [34] suggested that the improvement of adiponectin levels is highly associated with a loss in both SAT and VAT induced by exercise. A recent study showed that aerobic exercise alone or combined with diet resulted in a significant increase in circulating and adipose tissue adiponectin levels in obese women, regardless changes in body composition [35]. However, fat mass remained unchanged and adiponectin levels decreased in hyperleptinemic groups (OVG and OBG). Recently, another group reported an adiponectin concentration increase in a non-hyperleptinemic group only. The leptin concentration was positively correlated with adiponectin, as demonstrated in this study [36]. We suggested that the decrease in leptin concentration may be associated to adiponectin reduction.

Total cholesterol, LDL-c, HDL-c, and homocysteine are also associated with cardiovascular disease and low muscle strength [37]. Many studies demonstrated that LDL and HDL cholesterol levels improved after moderate or intense training [38]. In our study, participation in a structured physical program was not effective in reducing LDL-c and total cholesterol levels. The improvement of the lipid profile due to exercise depends on the intensity and frequency, the duration of each session, and the time spent on such a program. In addition, conflicting results concerning the effects of physical activity on the blood lipid profile is limited in individuals with normal values of lipid reference [39], as in the subjects investigated. In our study, 8% of the participants had high total cholesterol (> 240 mg/dl), 19% had low HDL-cholesterol (< 40 mg/dl) and 16% reported taking dyslipidemic medication only. The high prevalence of dyslipidemia in the OBG (54%) and OVG (39%) and low levels of volunteers taking dyslipidemia medications (16%) could have contributed to the opposite effect during the long-term study. A significant reduction of body mass could enhance beneficial changes in the blood lipid profile [40] that was not reported in our investigation. Nonetheless, improvements induced by exercise on the lipid profile appears to occur independently of any changes in body composition [41].

Elevated levels of muscle sympathetic nerve activity are associated with obesity-induced subclinical organ damage to the heart, even in the absence of hypertension [42]. In accordance with other studies, moderate exercise reduced diastolic blood pressure [38, 43]. Physical activity may alter vascular compliance due to diminished hormone release such as resistin, leptin, and IL-6 that could reduce SNS activity and peripheral resistance [3].

In conclusion, moderate exercise without calorie restriction had no effect on body composition; however, training demonstrated beneficial effects on the low-grade inflammatory state, decreasing leptin, resistin and IL-6 and cardiovascular clinical markers such as blood pressure and homocysteine, mainly in overweight and/or obese individuals.
